# Virtual reality reduces COVID-19 vaccine hesitancy in the wild: a randomized trial

**DOI:** 10.1038/s41598-022-08120-4

**Published:** 2022-03-17

**Authors:** Clara Vandeweerdt, Tiffany Luong, Michael Atchapero, Aske Mottelson, Christian Holz, Guido Makransky, Robert Böhm

**Affiliations:** 1 Department of Psychology, University of Copenhagen, Copenhagen, Denmark; 2 Department of Political Science, University of Copenhagen, Copenhagen, Denmark; 3 Department of Computer Science, ETH Zürich, Zurich, Switzerland; 4 IT University of Copenhagen, Copenhagen, Denmark; 5 Faculty of Psychology, University of Vienna, Vienna, Austria; 6 Copenhagen Center for Social Data Science (SODAS), University of Copenhagen, Copenhagen, Denmark

**Keywords:** Preventive medicine, Human behaviour

## Abstract

Vaccine hesitancy poses one of the largest threats to global health. Informing people about the collective benefit of vaccination has great potential in increasing vaccination intentions. This research investigates the potential for engaging experiences in immersive virtual reality (VR) to strengthen participants’ understanding of community immunity, and therefore, their intention to get vaccinated. In a pre-registered lab-in-the-field intervention study, participants were recruited in a public park (tested: $n = 232$, analyzed: $n = 222$). They were randomly assigned to experience the collective benefit of community immunity in a gamified immersive virtual reality environment ($\frac{2}{3}$ of sample), or to receive the same information via text and images ($\frac{1}{3}$ of sample). Before and after the intervention, participants indicated their intention to take up a hypothetical vaccine for a new COVID-19 strain (0–100 scale) and belief in vaccination as a collective responsibility (1–7 scale). The study employs a crossover design (participants later received a second treatment), but the primary outcome is the effect of the first treatment on vaccination intention. After the VR treatment, for participants with less-than-maximal vaccination intention, intention increases by 9.3 points (95% CI: 7.0 to $11.5,\, p < 0.001$). The text-and-image treatment raises vaccination intention by 3.3 points (difference in effects: 5.8, 95% CI: 2.0 to $9.5,\, p = 0.003$). The VR treatment also increases collective responsibility by 0.82 points (95% CI: 0.37 to $1.27,\, p < 0.001$). The results suggest that VR interventions are an effective tool for boosting vaccination intention, and that they can be applied “in the wild”—providing a complementary method for vaccine advocacy.

## Introduction

Vaccination against most infectious diseases is an individual decision with positive externalities. That is, when individuals get vaccinated, they not only protect themselves, but typically also limit the probability that they will transmit the disease to others^1^. As such, even unvaccinated citizens can be indirectly protected from infection, known as *community immunity* or *herd immunity*^1^. With regard to the COVID-19 pandemic, it has been estimated that 60–90% of the population needs to be vaccinated (depending, for instance, on the vaccine’s efficacy) to stop the spread of SARS-CoV-2^2,3^. Therefore, vaccine hesitancy—defined as “the delay in acceptance or refusal of vaccination despite availability of vaccination services”^4^—is a key obstacle to ending the COVID-19 pandemic.

Vaccine hesitancy is complex and may be affected by several factors^5,6^: lack of confidence (i.e., the tendency to trust in the safety and effectiveness of vaccines and to trust health authorities and experts who develop, license, and recommend vaccines), complacency (i.e., low perceived risk of infectious diseases), constraints (i.e., structural or psychological barriers in daily life that make vaccination difficult or costly), calculation (i.e., the degree to which personal costs and benefits of vaccination are weighted), lack of collective responsibility (i.e., the willingness to protect others and eliminate infectious diseases), lack of compliance (i.e., the support for societal monitoring and sanctioning of people who are not vaccinated), and conspiracy (i.e., conspiracy thinking and belief in fake news related to vaccination). Accordingly, fully informed vaccination decisions require that people know and understand the individual costs and benefits of a vaccination, as well as its collective benefit.

In line with the assumption that people care not only about their own but also about others’ welfare, informing them about community immunity has sometimes (e.g.,^7^) but not always (e.g.,^8^) been shown to increase vaccination intentions (for a review, see^9^). Interactive simulations have been particularly effective in increasing vaccine intentions^10–12^, potentially because they are more engaging^13^ and, therefore, increase people’s learning motivation^10,14^. In other words, using novel technologies that help people to better understand the collective benefit of vaccination-and the impact that their own vaccination may have on (vulnerable) others—may be a promising strategy to increase collective responsibility and, in turn, decrease vaccine hesitancy^15^.

Building on these findings, in this study we investigate whether vaccination intention is increased by a gamified immersive VR experience showing how community immunity works. Immersive VR is a promising medium for health communication (cf.^16^), because compared to other media it facilitates a high level of presence (the feeling of being in the virtual environment)^17^ and agency (the psychological experience of controlling one’s own actions)^18^, which results in higher levels of enjoyment and engagement^19,20^. Still, it has only just started to be tested as a tool for vaccine advocacy, with one study showing no significant impact on vaccination intentions^21^, and a second study finding a noticeable effect^22^.

In our VR simulation, participants must either try not to infect other non-player characters in a virtual scene, or try not to get infected by them. All participants play two scenarios—starting with an environment in which few characters are vaccinated, followed by an environment where many characters are vaccinated. The simulation thus allows participants to experience community immunity from a first-person perspective, learning how much more slowly infection spreads when vaccination rates are high versus low. Moreover, by using gamification in an immersive VR simulation, participants are likely to be motivated and engaged with the learning content^18,23^. We compare the effectiveness of this simulation against a typical information treatment using text and images.

We hypothesized that:

1. Vaccination intention increases after the VR treatment.
2. Vaccination intention increases more after the VR treatment than after the text-and-image treatment.
3. Collective responsibility increases after the VR treatment.
4. Collective responsibility increases more after the VR than than after the text-and-image treatment.

## Method

The design and analysis plan of this randomized control trial was preregistered on 03/06/2021, prior to accessing any data. See https://osf.io/cjgfe/?view_only=ac57eafb9fe54ed8bcd9d3bee16cc8a4 for the anonmyized plan. Registration DOI is 10.17605/OSF.IO/WUFXK. Unless otherwise noted, all steps below follow the pre-registration plan. The full study procedure was approved by the Institutional Review Board at the Psychology Department, University of Copenhagen. The study was performed in accordance with the ethical standards of the Declaration of Helsinki (1964) and its subsequent amendments. Informed consent was obtained from all participants.

### Recruitment

Participants (tested: $n = 232$, analyzed: $n = 222$) were 207 passersby recruited in a public park in Copenhagen during the first weekend in June 2021, plus 15 passersby recruited one week earlier on campus at the University of Copenhagen. All adults with basic understanding of English were eligible. The sample size was determined by the number of passersby who agreed to participate during the pre-registered study period. Respondents (aged 18 to 63) participated in exchange for drinks and snacks. Table 1 contains key descriptive statistics.

**Table 1:** Characteristics of the analyzed study sample ($n=222$).

Sample characteristic	Result
Age	29.0 (9.1)
% Female	39%
% Vaccinated	16%
Previous VR experience (median)	1–3 times
Pre-treatment vaccination intention	65.8 (25.6)
Pre-treatment collective responsibility	6.0 (1.5)
% Max pre-treatment vaccination intention	12%
% Max pre-treatment collective responsibility	53%

Continuous variables are summarized as mean (standard deviation). Last two rows are % of respondents who had maximum values on the outcome measures before receiving any treatment.

### Design

**Figure 1:**
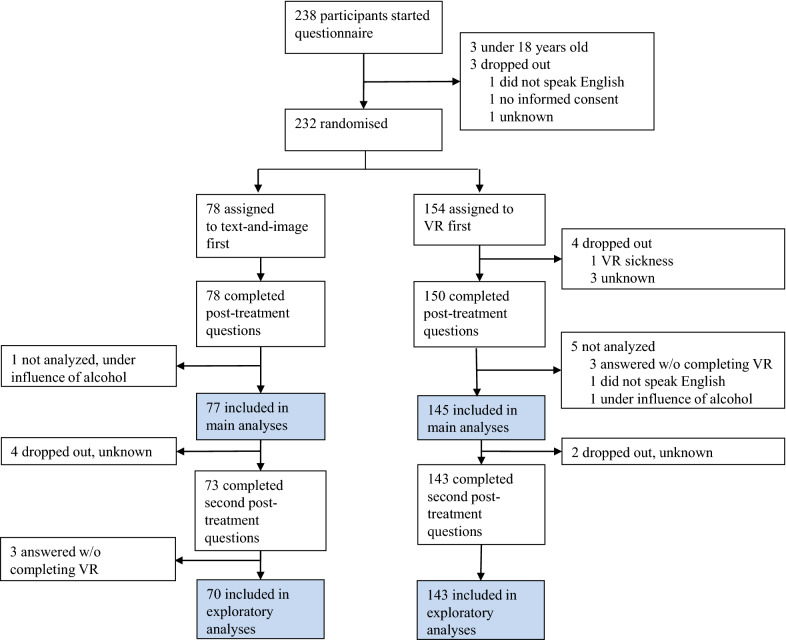
Trial profile showing flow of participants into treatment arms and analyses. Because participants largely self-administered the questionnaires and treatments, needing assistance only to start up the VR simulation, dropout reasons are sometimes unknown.

After filling out a pre-treatment questionnaire, $\frac{2}{3}$ of the participants were randomly assigned to the VR treatment. The other $\frac{1}{3}$ were randomly assigned to read a text and see images explaining community immunity. All participants then filled out a post-treatment questionnaire. Finally, all participants also received the treatment they had not been assigned to initially (VR or text-and-image), and filled out a second post-treatment questionnaire (crossover design). Figure 1 shows the trial profile.

Below, we detail the content of both treatments: the VR simulation and the text-and-image treatment.

#### VR

In the VR treatment, participants wore an Oculus Quest headset for a 5–10 min simulation developed at SIPLAB (ETH Zurich). They were embodied as an older character matching their gender. They were told that their character is vulnerable to COVID-19.

Participants were randomly assigned to one of two versions of the VR simulation. In the *avoid spreading* version, the player character (“avatar”) is already infected and the player must try not to infect others. In the *avoid infecting* version, the player character is uninfected and the player must try not to be exposed to infected characters.

In a first step, a tutorial showed the mechanics of the game. There were healthy-and-unvaccinated, infected (red clothing) and healthy-and-vaccinated (blue clothing) characters in the environment. Infected characters could spread the disease when coming too close to a healthy-and-unvaccinated character. Close contact was defined as a 2 m radius around the character.

In a second step, participants were tasked with crossing a busy square to reach a marked destination, while avoiding contact with the other 130 characters in the square. In the first scenario, they did so in an environment where 20% of the virtual characters were vaccinated. In the second scenario, they crossed the busy square again, but with 70% of the characters being vaccinated. Instructions clarified that the difference between the two scenarios was the avatars’ vaccination rate.

**Figure 2:**
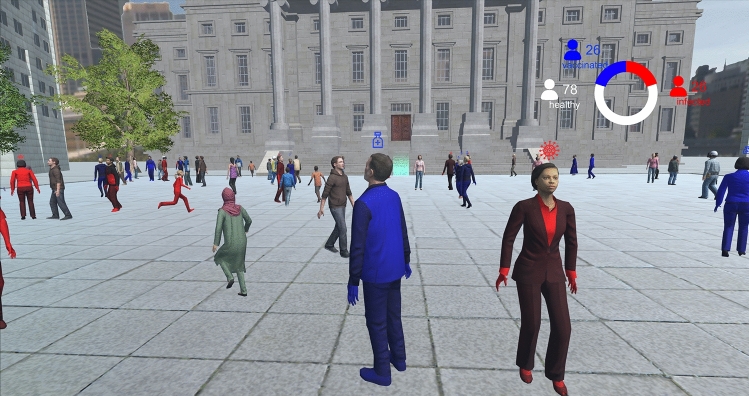
The busy square scene in VR, with feedback graph showing the number of infected, healthy and vaccinated characters.

When participants came into close contact with a character (infecting them or being exposed to their infection), they were made aware through graphics and haptic feedback (vibrating controllers). A small graph also helped them see how the disease spread between characters in the square, increasing the count of infected characters as they moved through the scenario. Figure 2 shows the square scene and spreading graph.

#### Text-and-image

The alternative treatment, using text and images, displayed the definition of community immunity by the US Centers for Disease Control and Prevention^24^, followed by two pictures (adapted from^10^) with captions. The pictures represented communities where few or many people are vaccinated. Captions explained that in a low-vaccination community, many healthy but unvaccinated people are at risk of infection. In a high-vaccination community, few are at risk.

Both the VR and text-and-image treatment ended with a brief summary, highlighting the takeaway message (“As you can see, when many people are vaccinated the virus does not spread as fast and it creates a world that is safer for everyone. You can see the difference in [...] the low and high vaccination scenarios”). A more detailed description of both treatments, including video, can be found in the study repository (https://osf.io/wufxk/?view_only=56e83d061c6d469fb6378d29c2940a4a).

### Randomization and masking

Simple randomization between treatment orderings (VR first or text first) happened within the Qualtrics survey software. Random assignment to a version of the VR simulation (avoid spreading or avoid infection) was tied to participants’ ID numbers (even or uneven), which were allocated consecutively to both VR-first and text-first participants. Experimenters only assisted participants in starting up the VR simulation. They were blind to both the treatment ordering and the VR simulation version that each participant was assigned to.

### Outcome measures

Two key measurements were taken before and after participants’ first treatment, as well as after their second treatment: First, vaccination intention for a hypothetical new COVID-19 strain was assessed (0–100 scale; adapted from^10^). This primary outcome measure was pretested in a pilot study available in the supplemental material. Second, seeing COVID-19 vaccination as a collective responsibility was assessed (1–7 scale)^5^. The supplemental material details the wording of these two items, and all other measures collected in the study.

All preregistered hypotheses are about the effect of the participants’ *first* treatment on the two outcome measures, either the VR treatment or the text-and-image treatment. The supplemental material section describes the models used to test these hypotheses; they are simple regressions of first differences in the outcome measures on medium of first treatment (VR or text).

## Results

**Figure 3:**
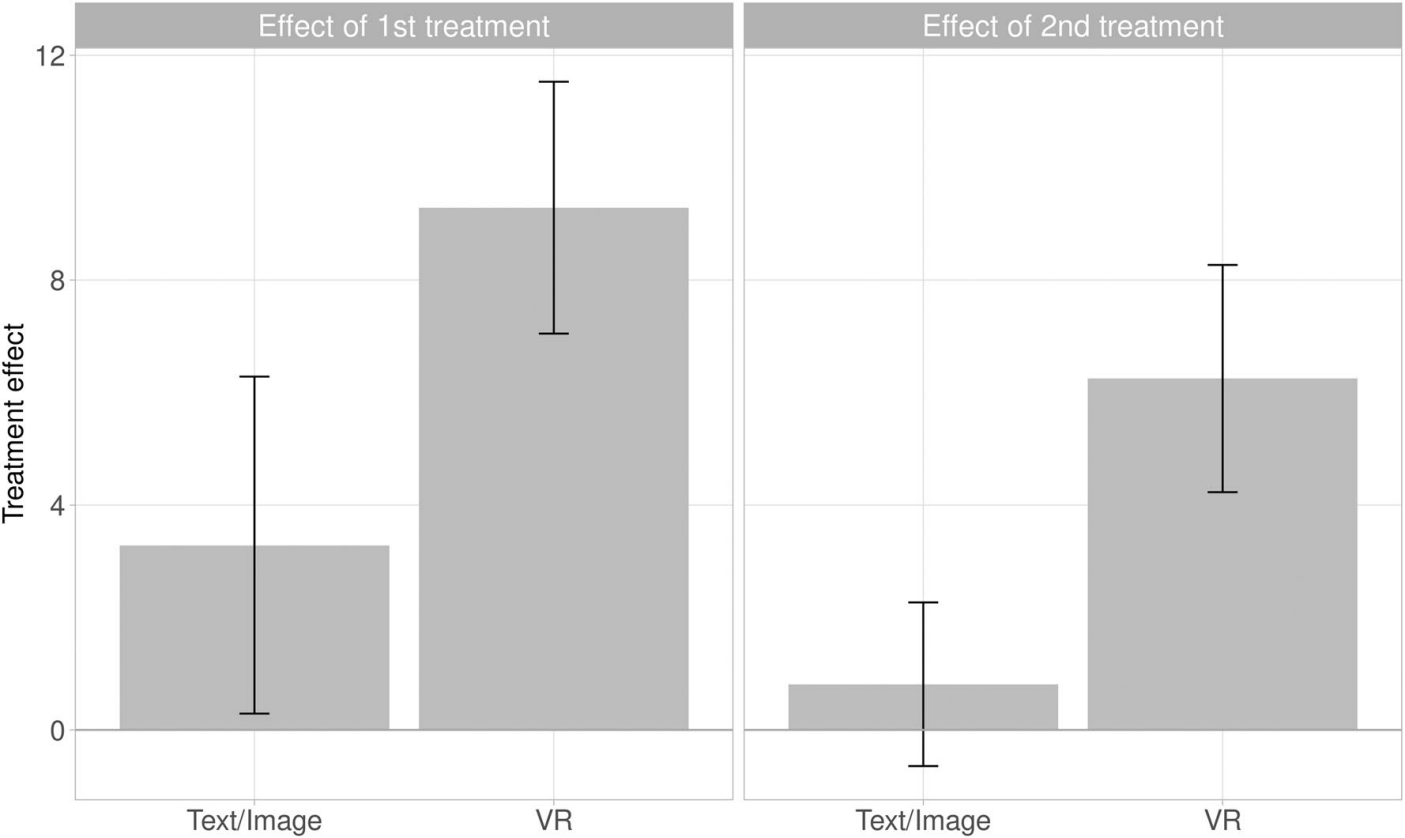
Average effect on vaccination intention of first treatment (*n* = 195, left panel) and second treatment (*n* = 189, right panel), leaving out participants with maximum pre-treatment vaccination intention. Error bars are 95% CIs.

Figure 3 (left panel) illustrates the effect of each treatment on vaccination intention as the difference between measurements before and after the first treatment. The supplemental material presents the full distribution of individual treatment effects, as well as analyses that do not exclude maximum-score participants.

Comparing vaccination intention between the pre-treatment and first post-treatment measure, for participants who did not already have maximally positive vaccination intention (*n* = 195), we find that the VR treatment increased vaccination intention by 9.3 points (95% CI: 7.0 to $11.5,\ p < 0.001$). The VR treatment is more effective than the text-and-image treatment, which only increases vaccination intention by 3.3 points (difference in effects: 5.8, 95% CI: 2.0 to $9.5,\ p = 0.003$).

Comparing collective responsibility pre- and post-treatment, for participants who did not already score the maximum on collective responsibility (*n* = 104), we find that the VR treatment increases collective responsibility by 0.82 points (95% CI: 0.37 to $1.27,\ p < 0.001$). The VR treatment is once again more effective than a text-and-image treatment, which increases collective responsibility by just 0.43 points, though the difference is not significant (difference in effects: 0.39, 95% CI: 0.36 to $1.14,\ p = 0.275$). The power to detect significant treatment differences is lower here, due to the smaller number of “moveable” participants with less-than-maximum perceptions of collective responsibility.

Further, we conducted an exploratory analysis on whether the VR treatment *further* increases vaccination intentions after a text-and-image treatment. Indeed, as shown in the right panel of Fig. 3, we find that for participants who experienced the text-and-image treatment first and did not have maximum pre-treatment vaccination intention, the subsequent VR treatment further increased vaccination intention by 6.3 points (95% CI: 4.2 to $8.3,\ p < 0.001$). In contrast, for those who received the text-and-image treatment after the VR treatment, there was no significant further increase in vaccination intention (effect: 0.8, 95% CI: $-0.6$ to $2.3,\ p = 0.309$).

We also explored any potential difference between the effectiveness of the two versions of the VR treatment: the one where participants avoided spreading COVID-19 and the one where they avoided infection with COVID-19. There is no difference in the effect of these two versions on either vaccination intention (difference in effects: 0.2, 95% CI: $-4.2$ to $47,\ p = 0.912$) or collective responsibility (difference in effects: $-0.003$, 95% CI: $-0.46$ to $0.45,\ p = 0.989$).

Finally, we asked all participants who completed the full study (*n* = 208) whether learning about community immunity via VR and text/pictures was fun, and whether they would like to receive more health communications via VR and text/pictures (1–5 scale). Compared to their ratings of text, participants rated VR as more fun (difference in means: 0.23, 95% CI: 0.11 to $0.35,\ p < 0.001$). There was no difference on how much participants wanted to receive future health communications via the two media (difference in means: $-0.06$, 95% CI: $-0.18$ to $0.05,\ p = 0.301$).

## Discussion

We provide seminal evidence that a first-person experience of vaccinations’ collective benefits in immersive VR can increase vaccination intentions. Further, the VR treatment is nearly three times more effective than communicating the same content via text and images. As the intended effect of both treatments is quite clear, the VR treatment’s greater effectiveness shows that its impact cannot be reduced to demand characteristics. This is further supported by the finding that adding the VR treatment after the text-and-image treatment further increased vaccination intention, whereas adding the text-and-image treatment after the VR treatment did not provide any further benefit.

Our results suggest that, due to the unique type of content it allows for, a VR intervention communicating about the collective benefit of vaccination can go beyond merely providing information. The results fit into a growing literature on the effectiveness of communicating about community immunity on vaccination intentions (see^9^ for a review) and builds on the finding that such interventions appear more effective when they are more engaging, such as via interactive simulations^10–12^, and when they elicit emotions, such as empathy^7^. As we demonstrate here, immersive VR is an effective alternative communication medium that can convey the collective benefit of vaccination in a highly engaging and emotional way.

There are several potential mechanisms for why the VR treatment leads to changes in behavioral intentions that deserve further investigation in future research. Our participants reported greater fun with the VR treatment than with the text-and-image treatment. Previous research has shown that immersive VR increases participants’ interest in the content domain^25^ and enjoyment^26^, which can increase attention and effort^18^ to understand a topic. Immersive simulations also induce a sense of presence^17^ and agency, which are essential for experiencing embodiment (the feeling of being in and controlling a virtual body)^27^. This can cause participants to associate negative and positive emotions with low and high vaccination rates, respectively. All of these features make it possible to create intense experiences of scenarios from another person’s perspective—increasing empathy with vulnerable others by allowing users to share their emotional processes^28^. Such effects are likely to further increase when elements of gamification are used^23^, as in the present study. Taken together, there are both cognitive and emotional features of the VR treatment that are likely influence behavioral intentions.

The current research has some limitations. Firstly, because is difficult to differentiate between mechanisms, it is currently unclear which aspects of the intervention may be modified by practitioners, and which ones must be kept. In future work, we will further investigate the mechanisms behind the effect of this intervention type, by developing more versions of the simulation, and measuring more intermediate variables (e.g., empathy).

Secondly, our outcome measures were vaccination intention and collective responsibility. We used established measures for these constructs and both have been linked to self-reported vaccine uptake^6,29^. Nevertheless, future research should investigate the effects on actual vaccination behaviour.

Thirdly, in contexts with limited funds and technological know-how, VR interventions are less feasible. Still, headsets have fallen dramatically in price, and they have become easier and more versatile in use—a trend that is expected to continue, making VR a more accessible option in the future.

Finally, since our sample was composed of passersby in a public park, people who had greater interest in VR may have been more likely to participate. Further, despite the fact that the study was advertised as a “VR experiment on COVID-19”, with no mention of vaccines or advocacy, it is possible that participants anticipated our objectives. This means that individuals with strong (anti-)vaccine beliefs may have declined to take part. Indeed, the strength of our invention lies in motivating the vaccine-hesitant to engage with vaccine-related content, rather than in persuading strong vaccine deniers.

Despite these limitations of the sample, showing the effectiveness of VR in increasing vaccination intentions “in the wild” indicates the generalizability of our findings to non-research settings. Moreover, the fact that VR attracts a specific audience may be beneficial. In fact, most volunteers in our study were younger adults—an age group that currently has lower COVID-19 vaccine uptake, including in countries where the vaccine is widely available^30,31^.

Although we found a substantial change in vaccination intentions, future research could develop an even more effective VR treatment. For example, subsequent versions may improve the experience’s narrative, or create more empathy with a main character who is especially vulnerable to the target disease. And while the present study focused on COVID-19 vaccination intentions, the VR treatment can also easily be adapted to, and tested for, different infectious diseases.

Our research contributes to a potential paradigm shift in health communication generally, and vaccine advocacy in particular. Finding novel methods to reduce vaccine hesitancy is critical^4,32,33^. Immersive, gamified VR provides a flexible tool to create more engaging and interactive learning experiences—alongside other media and technology, such as gamified apps and augmented reality. For vaccine and community immunity information in particular, it is crucial to reach and engage healthy members of the population (including young adults). Immersive VR has strong potential to complement more traditional communication channels and, therefore, contribute to decreasing the threat from infectious diseases.

## Data Availability

Anonymous individual participant data, plus analysis files a data dictionary with variable descriptions, are available to anyone from the study repository (https://osf.io/wufxk/?view_only=56e83d061c6d469fb6378d29c2940a4a). The repository also includes the study protocol, pre-analysis plan and informed consent form.
