# Supplementary material for: Virtual reality reduces COVID-19 vaccine hesitancy in the wild: a randomized trial
Source: Sci Rep. 2022 Mar 17;12:4593. doi: 10.1038/s41598-022-08120-4 (PMC8928717; doi:10.1038/s41598-022-08120-4)
Supplement: Supplementary file 1 — Supplementary Information. [file 41598_2022_8120_MOESM1_ESM.pdf]

Supplementary Material for Virtual reality reduces COVID-19 vaccine hesitancy in the wild: A randomized trial

Clara Vandeweerdt[1], Tiffany Luong[2], Michael Atchapero[1], Aske Mottelson[1], Christian Holz[2], Guido Makransky[1], and Robert Böhm[1,2,4]

[1]Department of Psychology, University of Copenhagen
[2]Department of Computer Science, ETH Zürich
[3]Department of Economics, University of Copenhagen
[4]Copenhagen Center for Social Data Science (SODAS), University of Copenhagen

August 2021

1 Pilot for vaccination intention item

To assess vaccination intention, we depart from the 1-100 "likelihood of getting vaccinated" item developed by [1], applied to the COVID-19 context. Acceptance of existing COVID-19 vaccines among our sample, collected in Denmark, was already high. In order to better observe the intervention effects without ceiling effects, we therefore adapted the measure to decrease the number of maximum-vaccination-intention responses. To do so, we created a hypothetical yet realistic and face-valid scenario:

> Suppose that in the future, a new strain of COVID-19 spreads, and that current vaccines are not effective against this strain. A new vaccine is developed against the new strain.
>
> The new vaccine seems effective and seems to have only mild side effects, but it has been tested on far fewer people.
>
> How likely would you be to get such a vaccine? (0 - would definitely not get it; 100 - would definitely get it)

We pre-tested this scenario in a pilot study with 125 UK citizens recruited via Prolific in May 2021. In this scenario, 24% of them had maximum vaccination intention, compared to 51% for the existing COVID-19 vaccines. Respondents

also found the scenario easy to imagine (4·4 on a 5-point scale), likely to happen (4·0) and realistic (4·1).

2 Question wordings

Superscripts indicate whether items are measured pre-treatment (1), after the first treatment (2), and/or after the second treatment (3).

Demographics^{1}

What is your age?

Have you already been vaccinated against COVID-19?

How many times have you used Virtual Reality before?

Vaccination intention^{123}

Suppose that in the future, a new strain of COVID-19 spreads, and that current vaccines are not effective against this strain. A new vaccine is developed against the new strain.

The new vaccine seems effective and seems to have only mild side effects, but it has been tested on far fewer people.

How likely would you be to get such a vaccine? (0 – would definitely not get it; 100 – would definitely get it)

Collective Responsibility^{123}

I see COVID-19 vaccination as a collective task against the spread of COVID-19.

(1 – strongly disagree; 7 – strongly agree)

Gender^{1}

At the start of the VR simulation, participants are asked to indicate their gender (male, female, other).

Presence and Embodiment[2 or 3]

(Administered once, just after the VR experience)

Presence (adapted from [2]):

- While I was in the square, I had a sense of "being there".
- I had a sense that I was interacting with other people in the square rather than a computer simulation.

(1 – strongly disagree; 5 – strongly agree)

Embodiment (adapted from [3])

I felt as if the virtual body I saw when I looked down was my body.

(1 – strongly disagree; 7 – strongly agree)

Media attitudes[3]

(order randomized: VR question first or text question first)

Next, we would like to hear your thoughts about the Virtual Reality simulation (busy square experience) that you saw in this study. How much do you agree with the following statements?

- It is fun to use a VR simulation to learn about the benefits of vaccines
- I would like to receive more health communication through Virtual Reality

Next, we would like to hear your thoughts about the text/picture communication (panels with colored dots) that you saw in this study. How much do you agree with the following statements?

- It is fun to use text and pictures to learn about the benefits of vaccines
- I would like to receive more health communication through text and pictures

3 Models for hypothesis testing

The pseudo-R code below shows the models that we use to test the preregistered hypotheses. Models focus on the change in vaccination intention and in collective responsibility after the first treatment, compared to before.

Model 1:

```
lm(vaccine_post_pre ~ textfirst, data=d[d$vaccine_pre != 100,])
```

Model 2:

```
lm(colres_post_pre ~ textfirst, data=d[d$colres_pre != 7,])
```

The intercepts of these models are used to estimate the effect of the VR treatment. That is, they estimate the size of the change in attitudes after the first treatment, for participants taking the VR treatment as their first treatment. The coefficients of `textfirst` compare the effect of the VR treatment to the effect of the text treatment. In both models, the sum of the intercept and `textfirst` coefficient shows the effect of the test-and-image treatment.

For the full analysis code, including exploratory analyses, see the study repository (`https://osf.io/wufxk/?view_only=56e83d061c6d469fb6378d29c2940a4a`).

4 Individual treatment effects

Figure S1 shows the individual treatment effects of the text-and-image treatment, VR, and their combination (with either text-and-image or VR coming first). These are measured as vaccine intention after treatment (or after both treatments), minus vaccine intention before treatment. As the Figure illustrates, negative treatment effects are quite rare. Most participants experience modest positive treatment effects (or no effect).

5 Including participants with maximum scores

Participants with maximum (i.e. 100) pre-treatment vaccination intention largely did not decrease. Among these 27 participants, 22 still had maximum vaccination intention after the first treatment. Average vaccination intention for these participants was 93.2 after the first treatment, with the drop being largely driven by a single participant whose intention went down to 0. In the full sample (including these participants), the effect of VR is 6.9 (95% CI: 4.5 to 9.3, $p < 0.001$). It is stronger than the effect of text and images by 4.1 points (95% CI: -8.2 to -0.08, $p < 0.05$). This analysis is exploratory and not preregistered.

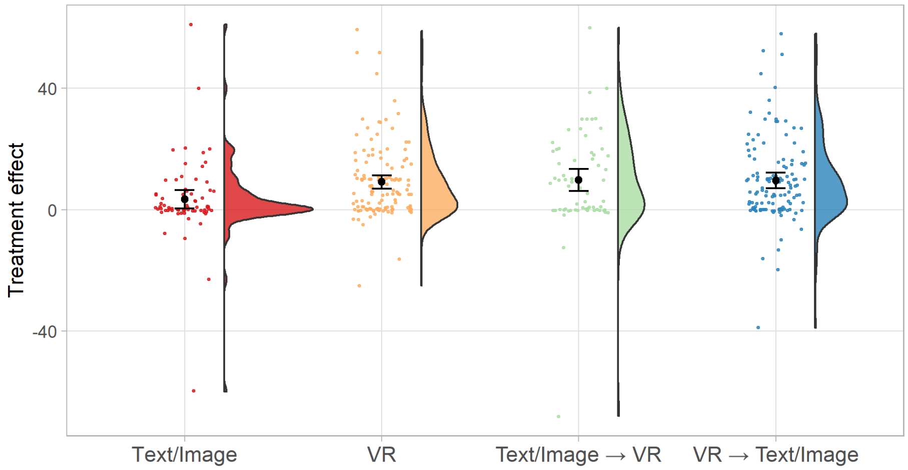

Figure S1: Individual-level change in vaccination intention from pre-treatment measurement, after first treatment (n = 195, first two columns) and after both treatments (n = 189, last two columns), leaving out participants with maximum pre-treatment vaccination intention. Colored dots are participants; colored graphs are distributions of individual treatment effects. Large black dots are average treatment effects with their 95% CIs.

References

[1] Cornelia Betsch, Robert Böhm, Lars Korn, and Cindy Holtmann. On the benefits of explaining herd immunity in vaccine advocacy. *Nature human behaviour*, 1(3):1–6, 2017.

[2] Guido Makransky, Lau Lilleholt, and Anders Aaby. Development and validation of the multimodal presence scale for virtual reality environments: A confirmatory factor analysis and item response theory approach. *Computers in Human Behavior*, 72:276–285, 2017.

[3] Mar Gonzalez-Franco and Tabitha C Peck. Avatar embodiment. towards a standardized questionnaire. *Frontiers in Robotics and AI*, 5:74, 2018.
